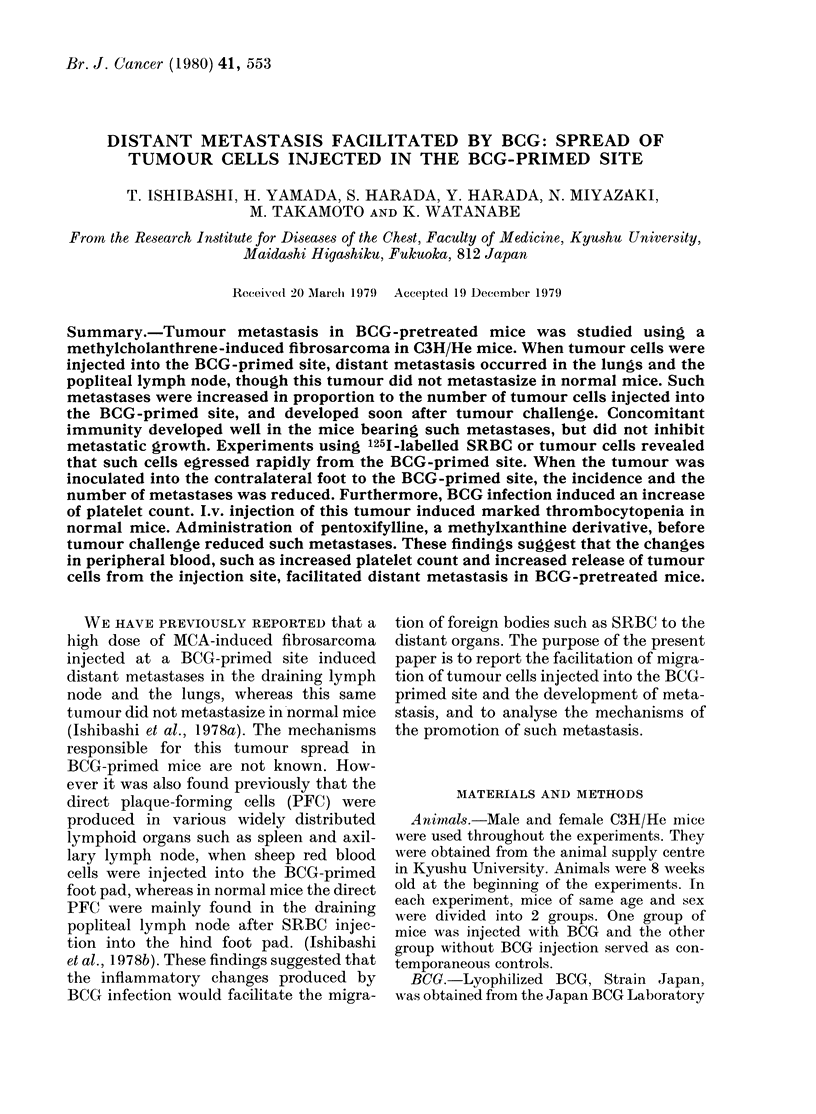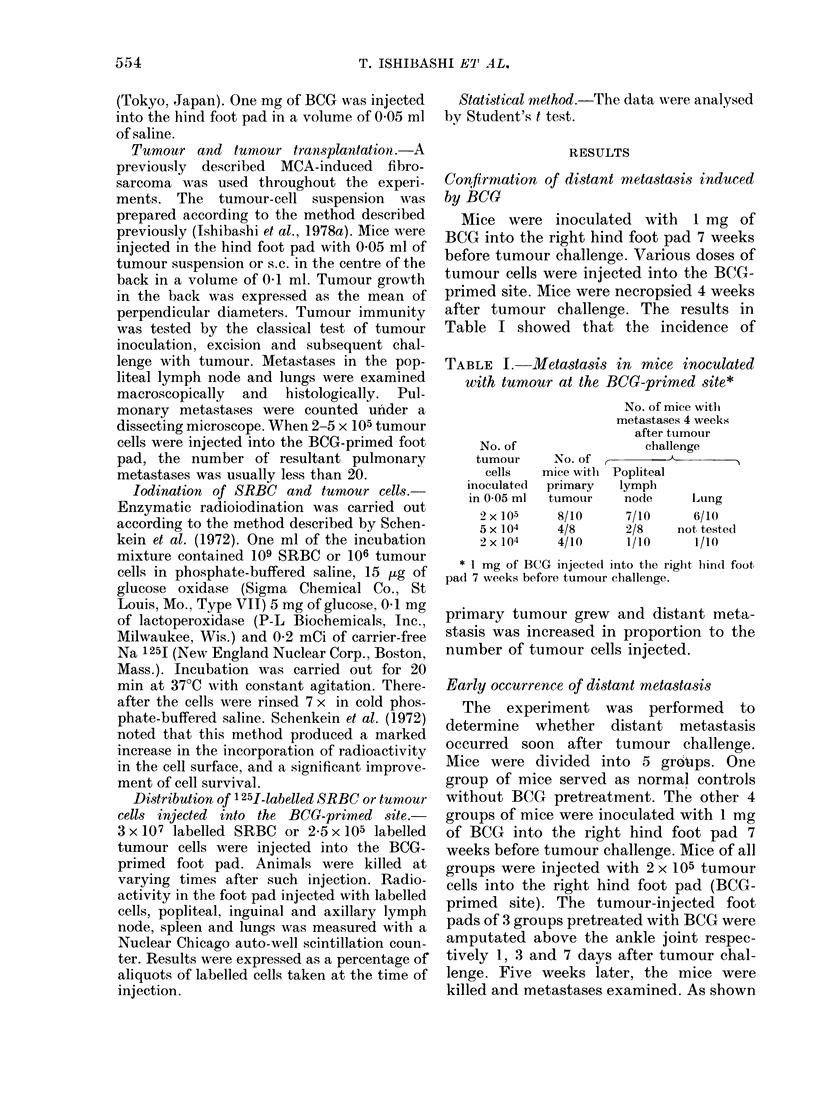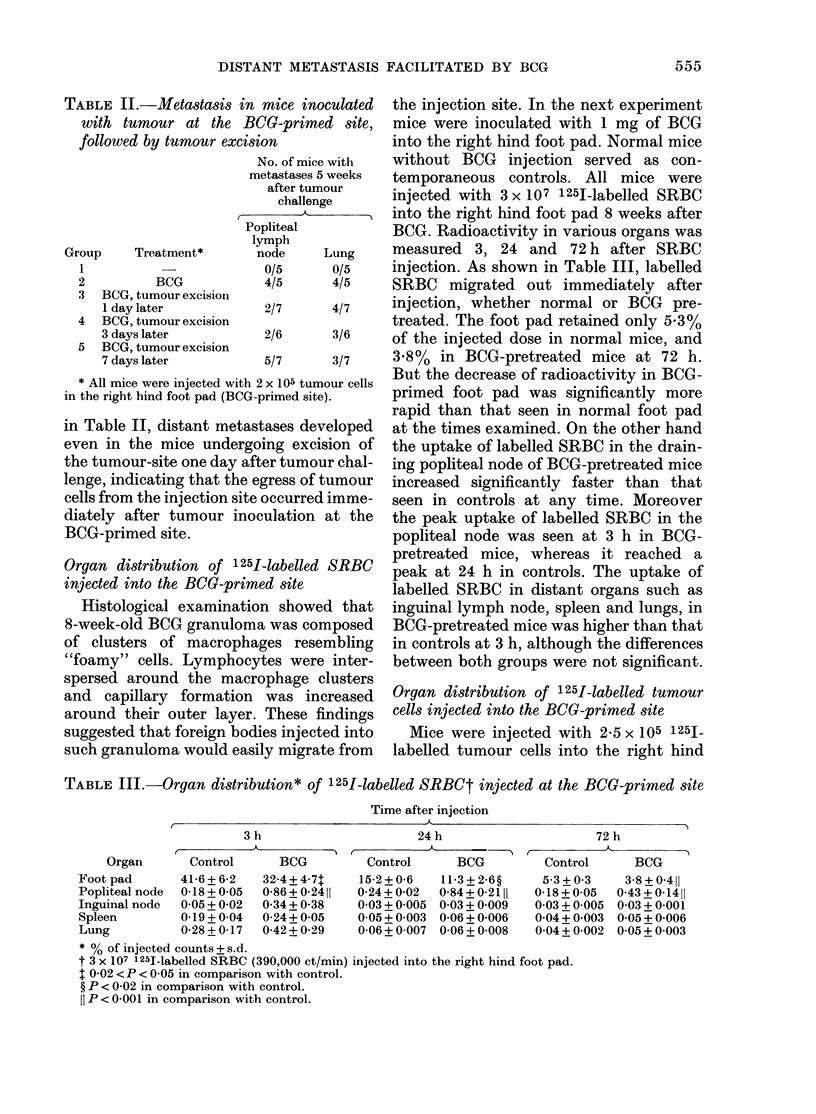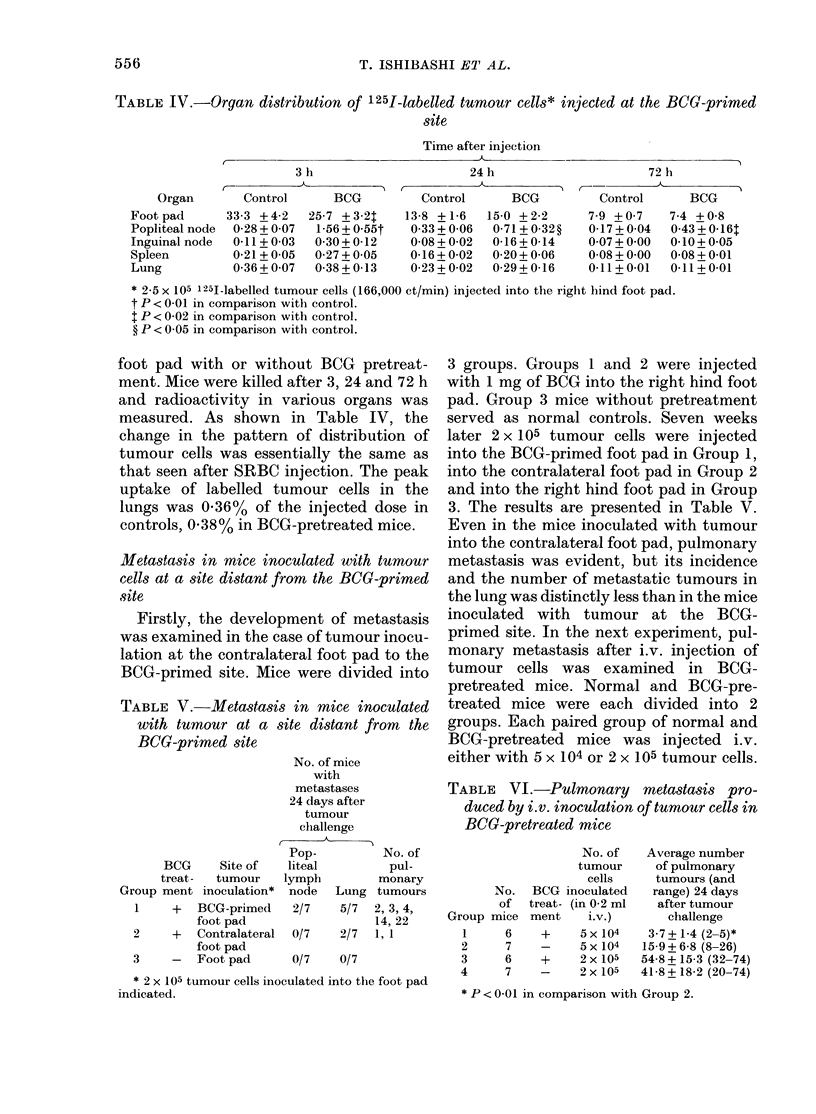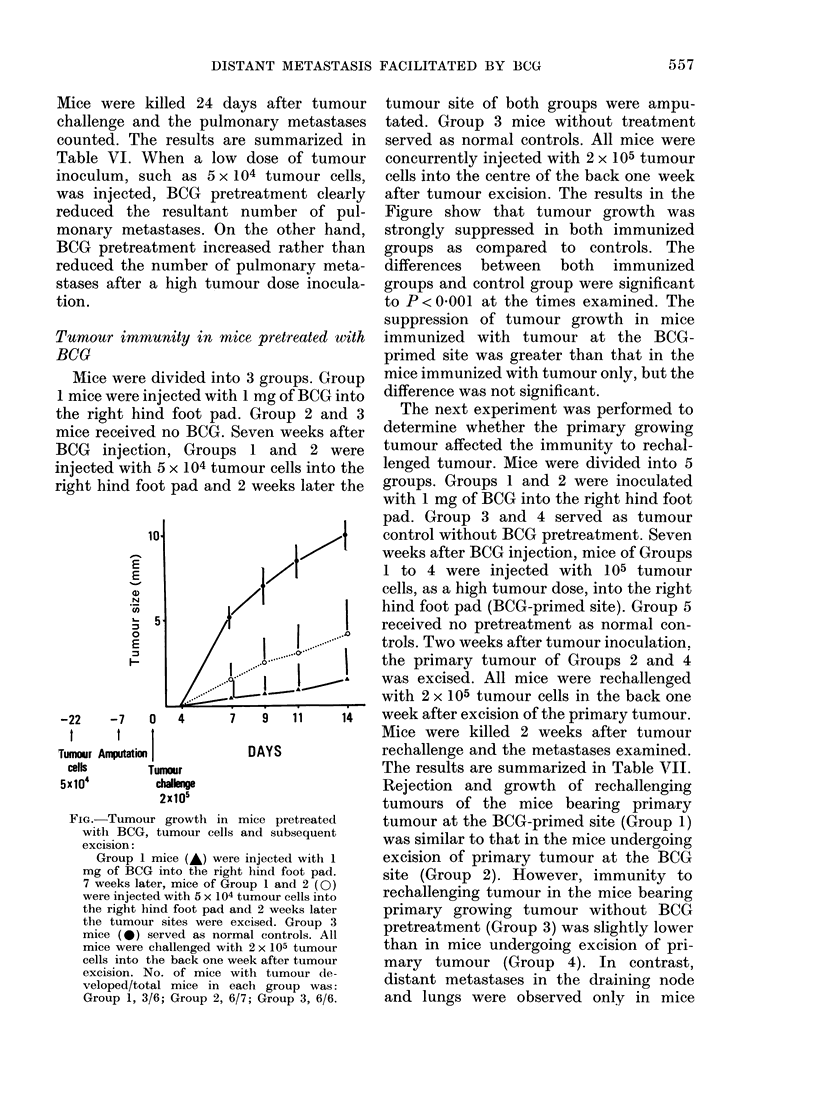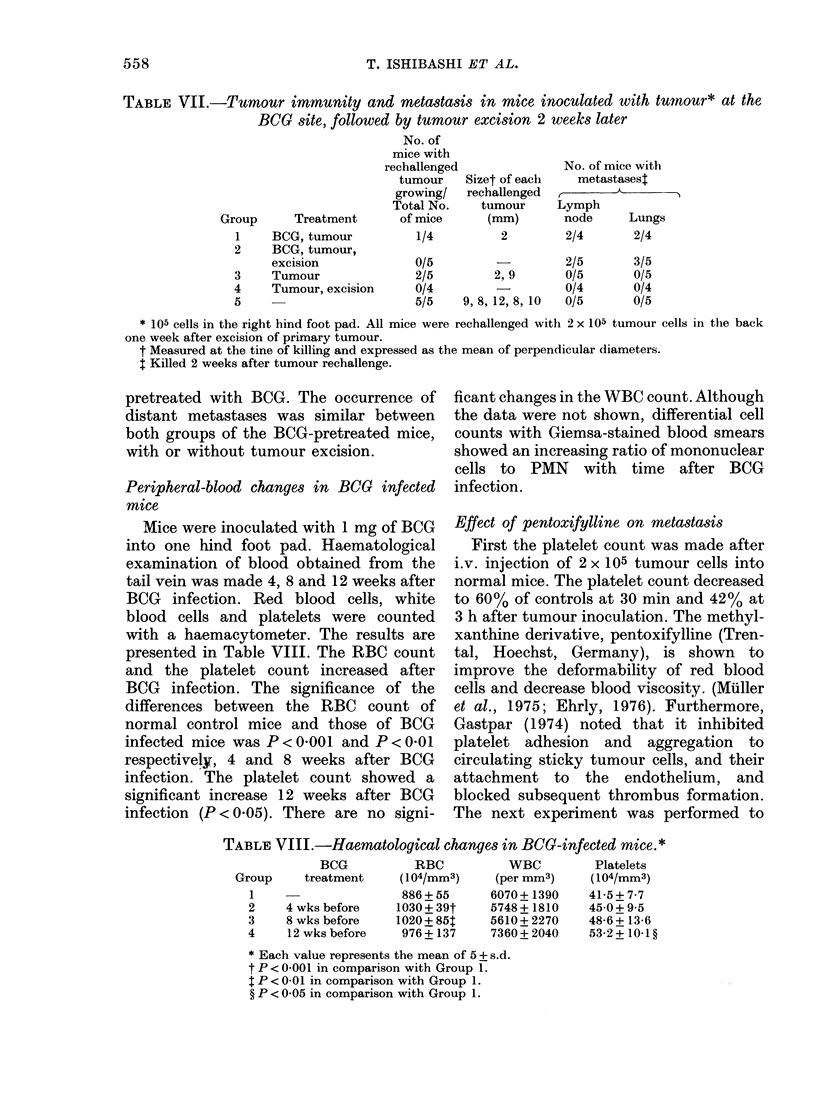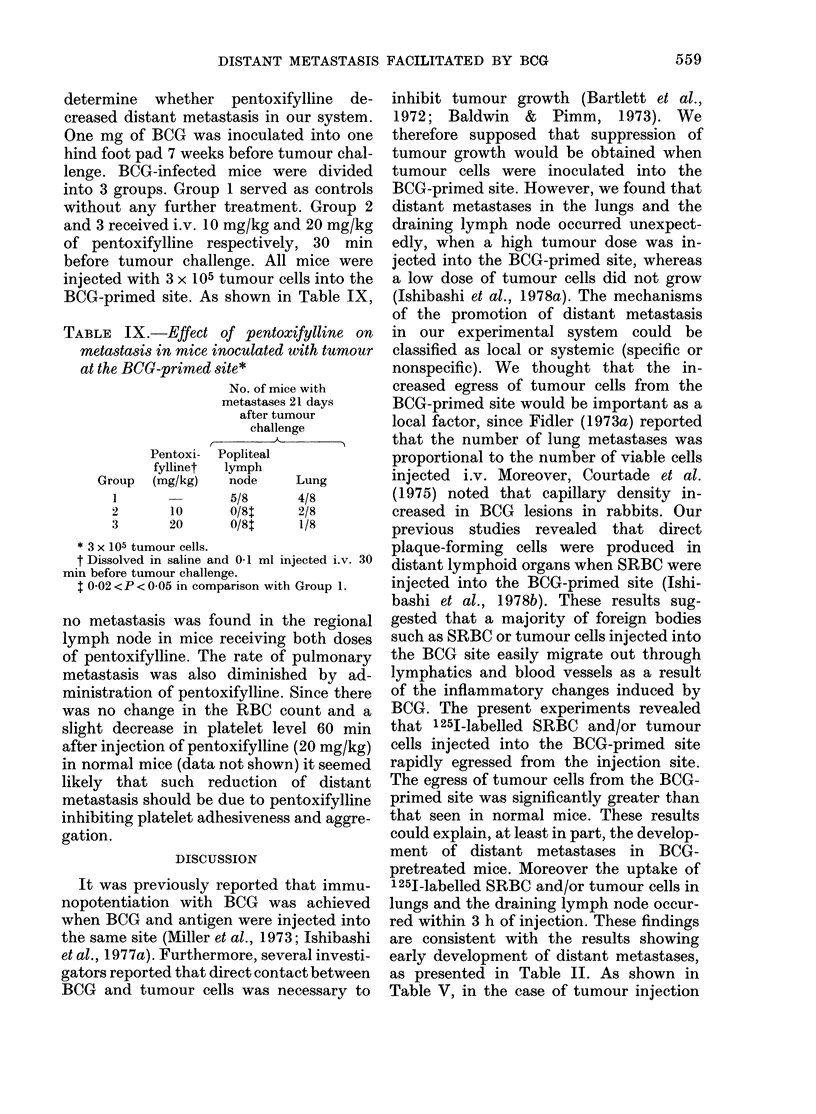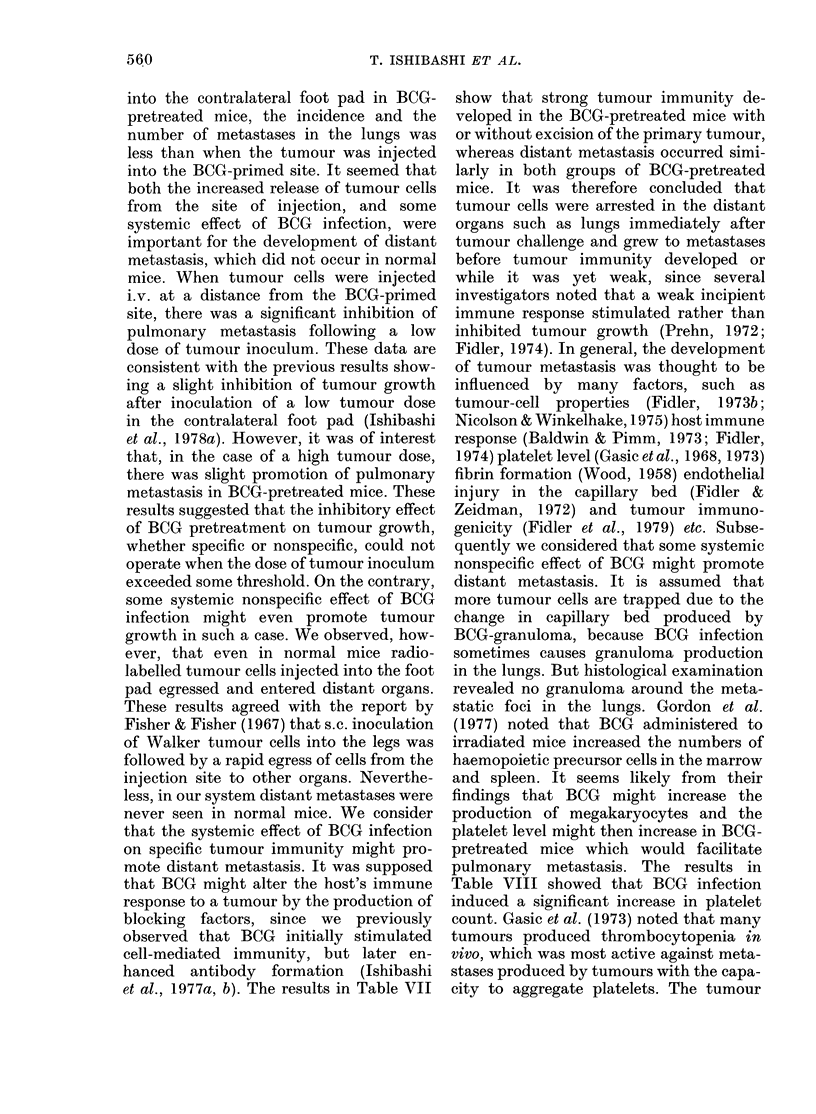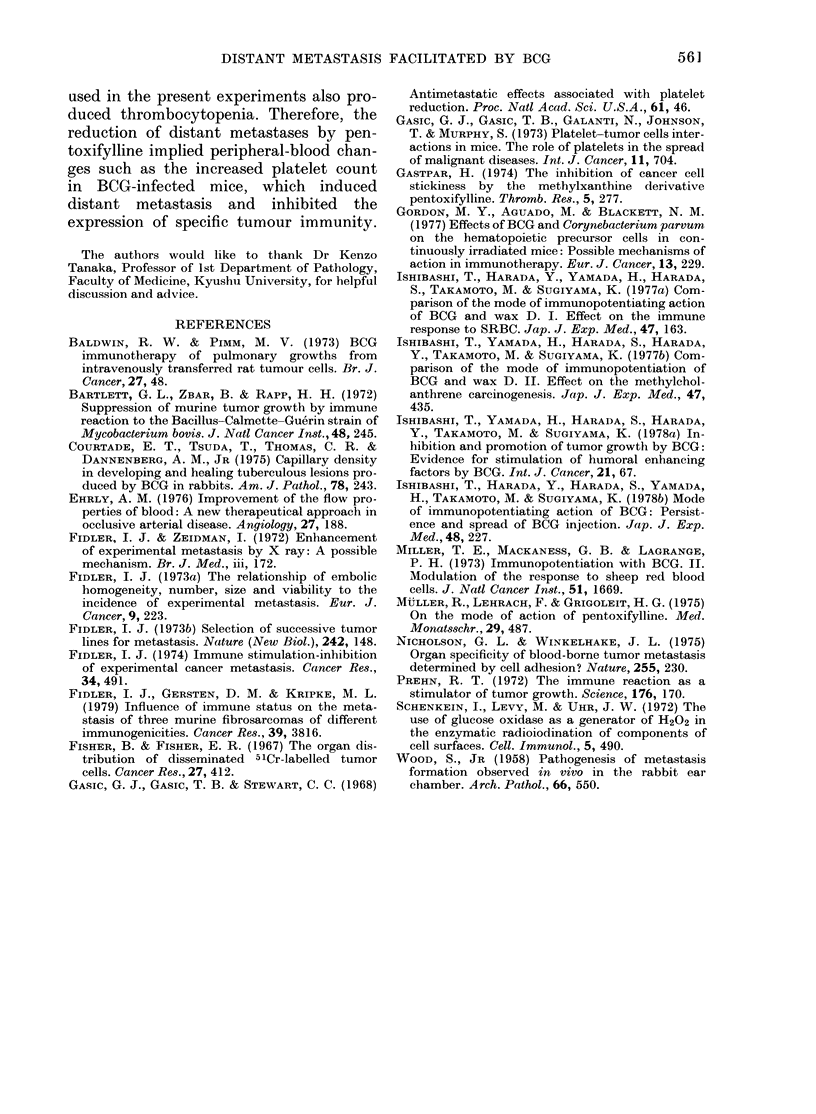# Distant metastasis facilitated by BCG: spread of tumour cells injected in the BCG-primed site.

**DOI:** 10.1038/bjc.1980.97

**Published:** 1980-04

**Authors:** T. Ishibashi, H. Yamada, S. Harada, Y. Harada, N. Miyazaki, M. Takamoto, K. Watanabe

## Abstract

Tumour metastasis in BCG-pretreated mice was studied using a methylcholanthrene-induced fibrosarcoma in C3H/He mice. When tumour cells were injected into the BCG-primed site, distant metastasis occurred in the lungs and the popliteal lymph node, through this tumour did not metastasize in normal mice. Such metastases were increased in proportion to the number of tumour cells injected into the BCG-primed site, and developed soon after tumour challenge. Concomitant immunity developed well in the mice bearing such metastases, but did not inhibit metastatic growth. Experiments using 125I-labelled SRBC or tumour cells revealed that such cells egressed rapidly from the BCG-primed site. When the tumour was inoculated into the contralateral foot to the BCG-primed site, the incidence and the number of metastases was reduced. Furthermore, BCG infection induced an increase of platelet count. I.v. injection of this tumour induced marked thrombocytopenia in normal mice. Administration of pentoxifylline, a methylxanthine derivative before tumour challenge reduced such metastases. These findings suggest that the changes in peripheral blood, such as increased platelet count and increased release of tumour cells from the injection site, facilitated distant metastasis in BCG-pretreated mice.


*Br. J. Cancer* (1980) **41**, 553

### DISTANT METASTASIS FACILITATED BY BCG: SPREAD OF TUMOUR CELLS INJECTED IN THE BCG-PRIMED SITE

T. ISHIBASHI, H. YAMADA, S. HARADA, Y. HARADA, N. MIYAZAKI, M. TAKAMOTO AND K. WATANABE

*From the Research Institute for Diseases of the Chest, Faculty of Medicine, Kyushu University, Maidashi Higashiku, Fukuoka, 812 Japan*

Received 20 March 1979 Accepted 19 December 1979

**Summary.—Tumour metastasis in BCG-pretreated mice was studied using a methylcholanthrene-induced fibrosarcoma in C3H/He mice. When tumour cells were injected into the BCG-primed site, distant metastasis occurred in the lungs and the popliteal lymph node, though this tumour did not metastasize in normal mice. Such metastases were increased in proportion to the number of tumour cells injected into the BCG-primed site, and developed soon after tumour challenge. Concomitant immunity developed well in the mice bearing such metastases, but did not inhibit metastatic growth. Experiments using $^{125}$I-labelled SRBC or tumour cells revealed that such cells egressed rapidly from the BCG-primed site. When the tumour was inoculated into the contralateral foot to the BCG-primed site, the incidence and the number of metastases was reduced. Furthermore, BCG infection induced an increase of platelet count. I.v. injection of this tumour induced marked thrombocytopenia in normal mice. Administration of pentoxifylline, a methylxanthine derivative, before tumour challenge reduced such metastases. These findings suggest that the changes in peripheral blood, such as increased platelet count and increased release of tumour cells from the injection site, facilitated distant metastasis in BCG-pretreated mice.**

WE HAVE PREVIOUSLY REPORTED that a high dose of MCA-induced fibrosarcoma injected at a BCG-primed site induced distant metastases in the draining lymph node and the lungs, whereas this same tumour did not metastasize in normal mice (Ishibashi *et al.*, 1978*a*). The mechanisms responsible for this tumour spread in BCG-primed mice are not known. However it was also found previously that the direct plaque-forming cells (PFC) were produced in various widely distributed lymphoid organs such as spleen and axillary lymph node, when sheep red blood cells were injected into the BCG-primed foot pad, whereas in normal mice the direct PFC were mainly found in the draining popliteal lymph node after SRBC injection into the hind foot pad. (Ishibashi *et al.*, 1978*b*). These findings suggested that the inflammatory changes produced by BCG infection would facilitate the migration of foreign bodies such as SRBC to the distant organs. The purpose of the present paper is to report the facilitation of migration of tumour cells injected into the BCG-primed site and the development of metastasis, and to analyse the mechanisms of the promotion of such metastasis.

### MATERIALS AND METHODS

*Animals.*—Male and female C3H/He mice were used throughout the experiments. They were obtained from the animal supply centre in Kyushu University. Animals were 8 weeks old at the beginning of the experiments. In each experiment, mice of same age and sex were divided into 2 groups. One group of mice was injected with BCG and the other group without BCG injection served as contemporaneous controls.

*BCG.*—Lyophilized BCG, Strain Japan, was obtained from the Japan BCG Laboratory

(Tokyo, Japan). One mg of BCG was injected into the hind foot pad in a volume of 0·05 ml of saline.

*Tumour and tumour transplantation.*—A previously described MCA-induced fibrosarcoma was used throughout the experiments. The tumour-cell suspension was prepared according to the method described previously (Ishibashi *et al.*, 1978*a*). Mice were injected in the hind foot pad with 0·05 ml of tumour suspension or s.c. in the centre of the back in a volume of 0·1 ml. Tumour growth in the back was expressed as the mean of perpendicular diameters. Tumour immunity was tested by the classical test of tumour inoculation, excision and subsequent challenge with tumour. Metastases in the popliteal lymph node and lungs were examined macroscopically and histologically. Pulmonary metastases were counted under a dissecting microscope. When $2–5 \times 10^5$ tumour cells were injected into the BCG-primed foot pad, the number of resultant pulmonary metastases was usually less than 20.

*Iodination of SRBC and tumour cells.*—Enzymatic radioiodination was carried out according to the method described by Schenkein *et al.* (1972). One ml of the incubation mixture contained $10^9$ SRBC or $10^6$ tumour cells in phosphate-buffered saline, 15 μg of glucose oxidase (Sigma Chemical Co., St Louis, Mo., Type VII) 5 mg of glucose, 0·1 mg of lactoperoxidase (P-L Biochemicals, Inc., Milwaukee, Wis.) and 0·2 mCi of carrier-free Na $^{125}$I (New England Nuclear Corp., Boston, Mass.). Incubation was carried out for 20 min at 37°C with constant agitation. Thereafter the cells were rinsed 7 × in cold phosphate-buffered saline. Schenkein *et al.* (1972) noted that this method produced a marked increase in the incorporation of radioactivity in the cell surface, and a significant improvement of cell survival.

*Distribution of $^{125}$I-labelled SRBC or tumour cells injected into the BCG-primed site.*—$3 \times 10^7$ labelled SRBC or $2·5 \times 10^5$ labelled tumour cells were injected into the BCG-primed foot pad. Animals were killed at varying times after such injection. Radioactivity in the foot pad injected with labelled cells, popliteal, inguinal and axillary lymph node, spleen and lungs was measured with a Nuclear Chicago auto-well scintillation counter. Results were expressed as a percentage of aliquots of labelled cells taken at the time of injection.

*Statistical method.*—The data were analysed by Student's *t* test.

### RESULTS

### *Confirmation of distant metastasis induced by BCG*

Mice were inoculated with 1 mg of BCG into the right hind foot pad 7 weeks before tumour challenge. Various doses of tumour cells were injected into the BCG-primed site. Mice were necropsied 4 weeks after tumour challenge. The results in Table I showed that the incidence of primary tumour grew and distant metastasis was increased in proportion to the number of tumour cells injected.

TABLE I.—*Metastasis in mice inoculated with tumour at the BCG-primed site**

| No. of tumour cells inoculated in 0·05 ml | No. of mice with primary tumour | No. of mice with metastases 4 weeks after tumour challenge | |
|---|---|---|---|
| | | Popliteal lymph node | Lung |
| $2 \times 10^5$ | 8/10 | 7/10 | 6/10 |
| $5 \times 10^4$ | 4/8 | 2/8 | not tested |
| $2 \times 10^4$ | 4/10 | 1/10 | 1/10 |

* 1 mg of BCG injected into the right hind foot pad 7 weeks before tumour challenge.

### *Early occurrence of distant metastasis*

The experiment was performed to determine whether distant metastasis occurred soon after tumour challenge. Mice were divided into 5 groups. One group of mice served as normal controls without BCG pretreatment. The other 4 groups of mice were inoculated with 1 mg of BCG into the right hind foot pad 7 weeks before tumour challenge. Mice of all groups were injected with $2 \times 10^5$ tumour cells into the right hind foot pad (BCG-primed site). The tumour-injected foot pads of 3 groups pretreated with BCG were amputated above the ankle joint respectively 1, 3 and 7 days after tumour challenge. Five weeks later, the mice were killed and metastases examined. As shown

TABLE II.—*Metastasis in mice inoculated with tumour at the BCG-primed site, followed by tumour excision*

| Group | Treatment* | No. of mice with metastases 5 weeks after tumour challenge: Popliteal lymph node | Lung |
|---|---|---|---|
| 1 | — | 0/5 | 0/5 |
| 2 | BCG | 4/5 | 4/5 |
| 3 | BCG, tumour excision 1 day later | 2/7 | 4/7 |
| 4 | BCG, tumour excision 3 days later | 2/6 | 3/6 |
| 5 | BCG, tumour excision 7 days later | 5/7 | 3/7 |

* All mice were injected with $2 \times 10^5$ tumour cells in the right hind foot pad (BCG-primed site).

in Table II, distant metastases developed even in the mice undergoing excision of the tumour-site one day after tumour challenge, indicating that the egress of tumour cells from the injection site occurred immediately after tumour inoculation at the BCG-primed site.

*Organ distribution of $^{125}$I-labelled SRBC injected into the BCG-primed site*

Histological examination showed that 8-week-old BCG granuloma was composed of clusters of macrophages resembling "foamy" cells. Lymphocytes were interspersed around the macrophage clusters and capillary formation was increased around their outer layer. These findings suggested that foreign bodies injected into such granuloma would easily migrate from the injection site. In the next experiment mice were inoculated with 1 mg of BCG into the right hind foot pad. Normal mice without BCG injection served as contemporaneous controls. All mice were injected with $3 \times 10^7$ $^{125}$I-labelled SRBC into the right hind foot pad 8 weeks after BCG. Radioactivity in various organs was measured 3, 24 and 72 h after SRBC injection. As shown in Table III, labelled SRBC migrated out immediately after injection, whether normal or BCG pretreated. The foot pad retained only 5·3% of the injected dose in normal mice, and 3·8% in BCG-pretreated mice at 72 h. But the decrease of radioactivity in BCG-primed foot pad was significantly more rapid than that seen in normal foot pad at the times examined. On the other hand the uptake of labelled SRBC in the draining popliteal node of BCG-pretreated mice increased significantly faster than that seen in controls at any time. Moreover the peak uptake of labelled SRBC in the popliteal node was seen at 3 h in BCG-pretreated mice, whereas it reached a peak at 24 h in controls. The uptake of labelled SRBC in distant organs such as inguinal lymph node, spleen and lungs, in BCG-pretreated mice was higher than that in controls at 3 h, although the differences between both groups were not significant.

*Organ distribution of $^{125}$I-labelled tumour cells injected into the BCG-primed site*

Mice were injected with $2{\cdot}5 \times 10^5$ $^{125}$I-labelled tumour cells into the right hind

TABLE III.—*Organ distribution* of $^{125}$I-labelled SRBC† injected at the BCG-primed site*

| Organ | 3 h Control | 3 h BCG | 24 h Control | 24 h BCG | 72 h Control | 72 h BCG |
|---|---|---|---|---|---|---|
| Foot pad | 41·6 ± 6·2 | 32·4 ± 4·7‡ | 15·2 ± 0·6 | 11·3 ± 2·6§ | 5·3 ± 0·3 | 3·8 ± 0·4‖ |
| Popliteal node | 0·18 ± 0·05 | 0·86 ± 0·24‖ | 0·24 ± 0·02 | 0·84 ± 0·21‖ | 0·18 ± 0·05 | 0·43 ± 0·14‖ |
| Inguinal node | 0·05 ± 0·02 | 0·34 ± 0·38 | 0·03 ± 0·005 | 0·03 ± 0·009 | 0·03 ± 0·005 | 0·03 ± 0·001 |
| Spleen | 0·19 ± 0·04 | 0·24 ± 0·05 | 0·05 ± 0·003 | 0·06 ± 0·006 | 0·04 ± 0·003 | 0·05 ± 0·006 |
| Lung | 0·28 ± 0·17 | 0·42 ± 0·29 | 0·06 ± 0·007 | 0·06 ± 0·008 | 0·04 ± 0·002 | 0·05 ± 0·003 |

* % of injected counts ± s.d.
† $3 \times 10^7$ $^{125}$I-labelled SRBC (390,000 ct/min) injected into the right hind foot pad.
‡ $0{\cdot}02 < P < 0{\cdot}05$ in comparison with control.
§ $P < 0{\cdot}02$ in comparison with control.
‖ $P < 0{\cdot}001$ in comparison with control.

TABLE IV.—*Organ distribution of $^{125}$I-labelled tumour cells* injected at the BCG-primed site*

| | Time after injection | | | | | |
|---|---|---|---|---|---|---|
| | 3 h | | 24 h | | 72 h | |
| Organ | Control | BCG | Control | BCG | Control | BCG |
| Foot pad | 33·3 ± 4·2 | 25·7 ± 3·2‡ | 13·8 ± 1·6 | 15·0 ± 2·2 | 7·9 ± 0·7 | 7·4 ± 0·8 |
| Popliteal node | 0·28 ± 0·07 | 1·56 ± 0·55† | 0·33 ± 0·06 | 0·71 ± 0·32§ | 0·17 ± 0·04 | 0·43 ± 0·16‡ |
| Inguinal node | 0·11 ± 0·03 | 0·30 ± 0·12 | 0·08 ± 0·02 | 0·16 ± 0·14 | 0·07 ± 0·00 | 0·10 ± 0·05 |
| Spleen | 0·21 ± 0·05 | 0·27 ± 0·05 | 0·16 ± 0·02 | 0·20 ± 0·06 | 0·08 ± 0·00 | 0·08 ± 0·01 |
| Lung | 0·36 ± 0·07 | 0·38 ± 0·13 | 0·23 ± 0·02 | 0·29 ± 0·16 | 0·11 ± 0·01 | 0·11 ± 0·01 |

* 2·5 × 10$^5$ $^{125}$I-labelled tumour cells (166,000 ct/min) injected into the right hind foot pad.
† $P < 0·01$ in comparison with control.
‡ $P < 0·02$ in comparison with control.
§ $P < 0·05$ in comparison with control.

foot pad with or without BCG pretreatment. Mice were killed after 3, 24 and 72 h and radioactivity in various organs was measured. As shown in Table IV, the change in the pattern of distribution of tumour cells was essentially the same as that seen after SRBC injection. The peak uptake of labelled tumour cells in the lungs was 0·36% of the injected dose in controls, 0·38% in BCG-pretreated mice.

*Metastasis in mice inoculated with tumour cells at a site distant from the BCG-primed site*

Firstly, the development of metastasis was examined in the case of tumour inoculation at the contralateral foot pad to the BCG-primed site. Mice were divided into 3 groups. Groups 1 and 2 were injected with 1 mg of BCG into the right hind foot pad. Group 3 mice without pretreatment served as normal controls. Seven weeks later 2 × 10$^5$ tumour cells were injected into the BCG-primed foot pad in Group 1, into the contralateral foot pad in Group 2 and into the right hind foot pad in Group 3. The results are presented in Table V. Even in the mice inoculated with tumour into the contralateral foot pad, pulmonary metastasis was evident, but its incidence and the number of metastatic tumours in the lung was distinctly less than in the mice inoculated with tumour at the BCG-primed site. In the next experiment, pulmonary metastasis after i.v. injection of tumour cells was examined in BCG-pretreated mice. Normal and BCG-pretreated mice were each divided into 2 groups. Each paired group of normal and BCG-pretreated mice was injected i.v. either with 5 × 10$^4$ or 2 × 10$^5$ tumour cells.

TABLE V.—*Metastasis in mice inoculated with tumour at a site distant from the BCG-primed site*

| | | | No. of mice with metastases 24 days after tumour challenge | | |
|---|---|---|---|---|---|
| Group | BCG treatment | Site of tumour inoculation* | Popliteal lymph node | Lung | No. of pulmonary tumours |
| 1 | + | BCG-primed foot pad | 2/7 | 5/7 | 2, 3, 4, 14, 22 |
| 2 | + | Contralateral foot pad | 0/7 | 2/7 | 1, 1 |
| 3 | − | Foot pad | 0/7 | 0/7 | |

* 2 × 10$^5$ tumour cells inoculated into the foot pad indicated.

TABLE VI.—*Pulmonary metastasis produced by i.v. inoculation of tumour cells in BCG-pretreated mice*

| Group | No. of mice | BCG treatment | No. of tumour cells inoculated (in 0·2 ml i.v.) | Average number of pulmonary tumours (and range) 24 days after tumour challenge |
|---|---|---|---|---|
| 1 | 6 | + | 5 × 10$^4$ | 3·7 ± 1·4 (2–5)* |
| 2 | 7 | − | 5 × 10$^4$ | 15·9 ± 6·8 (8–26) |
| 3 | 6 | + | 2 × 10$^5$ | 54·8 ± 15·3 (32–74) |
| 4 | 7 | − | 2 × 10$^5$ | 41·8 ± 18·2 (20–74) |

* $P < 0·01$ in comparison with Group 2.

Mice were killed 24 days after tumour challenge and the pulmonary metastases counted. The results are summarized in Table VI. When a low dose of tumour inoculum, such as $5 \times 10^4$ tumour cells, was injected, BCG pretreatment clearly reduced the resultant number of pulmonary metastases. On the other hand, BCG pretreatment increased rather than reduced the number of pulmonary metastases after a high tumour dose inoculation.

*Tumour immunity in mice pretreated with BCG*

Mice were divided into 3 groups. Group 1 mice were injected with 1 mg of BCG into the right hind foot pad. Group 2 and 3 mice received no BCG. Seven weeks after BCG injection, Groups 1 and 2 were injected with $5 \times 10^4$ tumour cells into the right hind foot pad and 2 weeks later the

FIG.—Tumour growth in mice pretreated with BCG, tumour cells and subsequent excision:

Group 1 mice (▲) were injected with 1 mg of BCG into the right hind foot pad. 7 weeks later, mice of Group 1 and 2 (○) were injected with $5 \times 10^4$ tumour cells into the right hind foot pad and 2 weeks later the tumour sites were excised. Group 3 mice (●) served as normal controls. All mice were challenged with $2 \times 10^5$ tumour cells into the back one week after tumour excision. No. of mice with tumour developed/total mice in each group was: Group 1, 3/6; Group 2, 6/7; Group 3, 6/6.

tumour site of both groups were amputated. Group 3 mice without treatment served as normal controls. All mice were concurrently injected with $2 \times 10^5$ tumour cells into the centre of the back one week after tumour excision. The results in the Figure show that tumour growth was strongly suppressed in both immunized groups as compared to controls. The differences between both immunized groups and control group were significant to $P < 0{\cdot}001$ at the times examined. The suppression of tumour growth in mice immunized with tumour at the BCG-primed site was greater than that in the mice immunized with tumour only, but the difference was not significant.

The next experiment was performed to determine whether the primary growing tumour affected the immunity to rechallenged tumour. Mice were divided into 5 groups. Groups 1 and 2 were inoculated with 1 mg of BCG into the right hind foot pad. Group 3 and 4 served as tumour control without BCG pretreatment. Seven weeks after BCG injection, mice of Groups 1 to 4 were injected with $10^5$ tumour cells, as a high tumour dose, into the right hind foot pad (BCG-primed site). Group 5 received no pretreatment as normal controls. Two weeks after tumour inoculation, the primary tumour of Groups 2 and 4 was excised. All mice were rechallenged with $2 \times 10^5$ tumour cells in the back one week after excision of the primary tumour. Mice were killed 2 weeks after tumour rechallenge and the metastases examined. The results are summarized in Table VII. Rejection and growth of rechallenging tumours of the mice bearing primary tumour at the BCG-primed site (Group 1) was similar to that in the mice undergoing excision of primary tumour at the BCG site (Group 2). However, immunity to rechallenging tumour in the mice bearing primary growing tumour without BCG pretreatment (Group 3) was slightly lower than in mice undergoing excision of primary tumour (Group 4). In contrast, distant metastases in the draining node and lungs were observed only in mice

TABLE VII.—*Tumour immunity and metastasis in mice inoculated with tumour* at the BCG site, followed by tumour excision 2 weeks later*

| Group | Treatment | No. of mice with rechallenged tumour growing/ Total No. of mice | Size† of each rechallenged tumour (mm) | No. of mice with metastases‡ Lymph node | Lungs |
|---|---|---|---|---|---|
| 1 | BCG, tumour | 1/4 | 2 | 2/4 | 2/4 |
| 2 | BCG, tumour, excision | 0/5 | — | 2/5 | 3/5 |
| 3 | Tumour | 2/5 | 2, 9 | 0/5 | 0/5 |
| 4 | Tumour, excision | 0/4 | — | 0/4 | 0/4 |
| 5 | — | 5/5 | 9, 8, 12, 8, 10 | 0/5 | 0/5 |

* $10^5$ cells in the right hind foot pad. All mice were rechallenged with $2 \times 10^5$ tumour cells in the back one week after excision of primary tumour.
† Measured at the tine of killing and expressed as the mean of perpendicular diameters.
‡ Killed 2 weeks after tumour rechallenge.

pretreated with BCG. The occurrence of distant metastases was similar between both groups of the BCG-pretreated mice, with or without tumour excision.

*Peripheral-blood changes in BCG infected mice*

Mice were inoculated with 1 mg of BCG into one hind foot pad. Haematological examination of blood obtained from the tail vein was made 4, 8 and 12 weeks after BCG infection. Red blood cells, white blood cells and platelets were counted with a haemacytometer. The results are presented in Table VIII. The RBC count and the platelet count increased after BCG infection. The significance of the differences between the RBC count of normal control mice and those of BCG infected mice was $P < 0{\cdot}001$ and $P < 0{\cdot}01$ respectively, 4 and 8 weeks after BCG infection. The platelet count showed a significant increase 12 weeks after BCG infection ($P < 0{\cdot}05$). There are no significant changes in the WBC count. Although the data were not shown, differential cell counts with Giemsa-stained blood smears showed an increasing ratio of mononuclear cells to PMN with time after BCG infection.

*Effect of pentoxifylline on metastasis*

First the platelet count was made after i.v. injection of $2 \times 10^5$ tumour cells into normal mice. The platelet count decreased to 60% of controls at 30 min and 42% at 3 h after tumour inoculation. The methylxanthine derivative, pentoxifylline (Trental, Hoechst, Germany), is shown to improve the deformability of red blood cells and decrease blood viscosity. (Müller *et al.*, 1975; Ehrly, 1976). Furthermore, Gastpar (1974) noted that it inhibited platelet adhesion and aggregation to circulating sticky tumour cells, and their attachment to the endothelium, and blocked subsequent thrombus formation. The next experiment was performed to

TABLE VIII.—*Haematological changes in BCG-infected mice.**

| Group | BCG treatment | RBC ($10^4$/mm$^3$) | WBC (per mm$^3$) | Platelets ($10^4$/mm$^3$) |
|---|---|---|---|---|
| 1 | — | 886 ± 55 | 6070 ± 1390 | 41·5 ± 7·7 |
| 2 | 4 wks before | 1030 ± 39† | 5748 ± 1810 | 45·0 ± 9·5 |
| 3 | 8 wks before | 1020 ± 85‡ | 5610 ± 2270 | 48·6 ± 13·6 |
| 4 | 12 wks before | 976 ± 137 | 7360 ± 2040 | 53·2 ± 10·1§ |

* Each value represents the mean of 5 ± s.d.
† $P < 0{\cdot}001$ in comparison with Group 1.
‡ $P < 0{\cdot}01$ in comparison with Group 1.
§ $P < 0{\cdot}05$ in comparison with Group 1.

determine whether pentoxifylline decreased distant metastasis in our system. One mg of BCG was inoculated into one hind foot pad 7 weeks before tumour challenge. BCG-infected mice were divided into 3 groups. Group 1 served as controls without any further treatment. Group 2 and 3 received i.v. 10 mg/kg and 20 mg/kg of pentoxifylline respectively, 30 min before tumour challenge. All mice were injected with $3 \times 10^5$ tumour cells into the BCG-primed site. As shown in Table IX,

TABLE IX.—*Effect of pentoxifylline on metastasis in mice inoculated with tumour at the BCG-primed site**

| | | No. of mice with metastases 21 days after tumour challenge | |
|---|---|---|---|
| Group | Pentoxifylline† (mg/kg) | Popliteal lymph node | Lung |
| 1 | — | 5/8 | 4/8 |
| 2 | 10 | 0/8‡ | 2/8 |
| 3 | 20 | 0/8‡ | 1/8 |

* $3 \times 10^5$ tumour cells.
† Dissolved in saline and 0·1 ml injected i.v. 30 min before tumour challenge.
‡ $0{\cdot}02 < P < 0{\cdot}05$ in comparison with Group 1.

no metastasis was found in the regional lymph node in mice receiving both doses of pentoxifylline. The rate of pulmonary metastasis was also diminished by administration of pentoxifylline. Since there was no change in the RBC count and a slight decrease in platelet level 60 min after injection of pentoxifylline (20 mg/kg) in normal mice (data not shown) it seemed likely that such reduction of distant metastasis should be due to pentoxifylline inhibiting platelet adhesiveness and aggregation.

### DISCUSSION

It was previously reported that immunopotentiation with BCG was achieved when BCG and antigen were injected into the same site (Miller *et al.*, 1973; Ishibashi *et al.*, 1977*a*). Furthermore, several investigators reported that direct contact between BCG and tumour cells was necessary to inhibit tumour growth (Bartlett *et al.*, 1972; Baldwin & Pimm, 1973). We therefore supposed that suppression of tumour growth would be obtained when tumour cells were inoculated into the BCG-primed site. However, we found that distant metastases in the lungs and the draining lymph node occurred unexpectedly, when a high tumour dose was injected into the BCG-primed site, whereas a low dose of tumour cells did not grow (Ishibashi *et al.*, 1978*a*). The mechanisms of the promotion of distant metastasis in our experimental system could be classified as local or systemic (specific or nonspecific). We thought that the increased egress of tumour cells from the BCG-primed site would be important as a local factor, since Fidler (1973*a*) reported that the number of lung metastases was proportional to the number of viable cells injected i.v. Moreover, Courtade *et al.* (1975) noted that capillary density increased in BCG lesions in rabbits. Our previous studies revealed that direct plaque-forming cells were produced in distant lymphoid organs when SRBC were injected into the BCG-primed site (Ishibashi *et al.*, 1978*b*). These results suggested that a majority of foreign bodies such as SRBC or tumour cells injected into the BCG site easily migrate out through lymphatics and blood vessels as a result of the inflammatory changes induced by BCG. The present experiments revealed that $^{125}$I-labelled SRBC and/or tumour cells injected into the BCG-primed site rapidly egressed from the injection site. The egress of tumour cells from the BCG-primed site was significantly greater than that seen in normal mice. These results could explain, at least in part, the development of distant metastases in BCG-pretreated mice. Moreover the uptake of $^{125}$I-labelled SRBC and/or tumour cells in lungs and the draining lymph node occurred within 3 h of injection. These findings are consistent with the results showing early development of distant metastases, as presented in Table II. As shown in Table V, in the case of tumour injection into the contralateral foot pad in BCG-pretreated mice, the incidence and the number of metastases in the lungs was less than when the tumour was injected into the BCG-primed site. It seemed that both the increased release of tumour cells from the site of injection, and some systemic effect of BCG infection, were important for the development of distant metastasis, which did not occur in normal mice. When tumour cells were injected i.v. at a distance from the BCG-primed site, there was a significant inhibition of pulmonary metastasis following a low dose of tumour inoculum. These data are consistent with the previous results showing a slight inhibition of tumour growth after inoculation of a low tumour dose in the contralateral foot pad (Ishibashi *et al.*, 1978*a*). However, it was of interest that, in the case of a high tumour dose, there was slight promotion of pulmonary metastasis in BCG-pretreated mice. These results suggested that the inhibitory effect of BCG pretreatment on tumour growth, whether specific or nonspecific, could not operate when the dose of tumour inoculum exceeded some threshold. On the contrary, some systemic nonspecific effect of BCG infection might even promote tumour growth in such a case. We observed, however, that even in normal mice radiolabelled tumour cells injected into the foot pad egressed and entered distant organs. These results agreed with the report by Fisher & Fisher (1967) that s.c. inoculation of Walker tumour cells into the legs was followed by a rapid egress of cells from the injection site to other organs. Nevertheless, in our system distant metastases were never seen in normal mice. We consider that the systemic effect of BCG infection on specific tumour immunity might promote distant metastasis. It was supposed that BCG might alter the host's immune response to a tumour by the production of blocking factors, since we previously observed that BCG initially stimulated cell-mediated immunity, but later enhanced antibody formation (Ishibashi *et al.*, 1977*a*, *b*). The results in Table VII show that strong tumour immunity developed in the BCG-pretreated mice with or without excision of the primary tumour, whereas distant metastasis occurred similarly in both groups of BCG-pretreated mice. It was therefore concluded that tumour cells were arrested in the distant organs such as lungs immediately after tumour challenge and grew to metastases before tumour immunity developed or while it was yet weak, since several investigators noted that a weak incipient immune response stimulated rather than inhibited tumour growth (Prehn, 1972; Fidler, 1974). In general, the development of tumour metastasis was thought to be influenced by many factors, such as tumour-cell properties (Fidler, 1973*b*; Nicolson & Winkelhake, 1975) host immune response (Baldwin & Pimm, 1973; Fidler, 1974) platelet level (Gasic *et al.*, 1968, 1973) fibrin formation (Wood, 1958) endothelial injury in the capillary bed (Fidler & Zeidman, 1972) and tumour immunogenicity (Fidler *et al.*, 1979) *etc.* Subsequently we considered that some systemic nonspecific effect of BCG might promote distant metastasis. It is assumed that more tumour cells are trapped due to the change in capillary bed produced by BCG-granuloma, because BCG infection sometimes causes granuloma production in the lungs. But histological examination revealed no granuloma around the metastatic foci in the lungs. Gordon *et al.* (1977) noted that BCG administered to irradiated mice increased the numbers of haemopoietic precursor cells in the marrow and spleen. It seems likely from their findings that BCG might increase the production of megakaryocytes and the platelet level might then increase in BCG-pretreated mice which would facilitate pulmonary metastasis. The results in Table VIII showed that BCG infection induced a significant increase in platelet count. Gasic *et al.* (1973) noted that many tumours produced thrombocytopenia *in vivo*, which was most active against metastases produced by tumours with the capacity to aggregate platelets. The tumour used in the present experiments also produced thrombocytopenia. Therefore, the reduction of distant metastases by pentoxifylline implied peripheral-blood changes such as the increased platelet count in BCG-infected mice, which induced distant metastasis and inhibited the expression of specific tumour immunity.

The authors would like to thank Dr Kenzo Tanaka, Professor of 1st Department of Pathology, Faculty of Medicine, Kyushu University, for helpful discussion and advice.

### REFERENCES

Baldwin, R. W. & Pimm, M. V. (1973) BCG immunotherapy of pulmonary growths from intravenously transferred rat tumour cells. *Br. J. Cancer*, **27**, 48.

Bartlett, G. L., Zbar, B. & Rapp, H. H. (1972) Suppression of murine tumor growth by immune reaction to the Bacillus–Calmette–Guérin strain of *Mycobacterium bovis*. *J. Natl Cancer Inst.*, **48**, 245.

Courtade, E. T., Tsuda, T., Thomas, C. R. & Dannenberg, A. M., Jr (1975) Capillary density in developing and healing tuberculous lesions produced by BCG in rabbits. *Am. J. Pathol.*, **78**, 243.

Ehrly, A. M. (1976) Improvement of the flow properties of blood: A new therapeutical approach in occlusive arterial disease. *Angiology*, **27**, 188.

Fidler, I. J. & Zeidman, I. (1972) Enhancement of experimental metastasis by X ray: A possible mechanism. *Br. J. Med.*, iii, 172.

Fidler, I. J. (1973*a*) The relationship of embolic homogeneity, number, size and viability to the incidence of experimental metastasis. *Eur. J. Cancer*, **9**, 223.

Fidler, I. J. (1973*b*) Selection of successive tumor lines for metastasis. *Nature* (*New Biol.*), **242**, 148.

Fidler, I. J. (1974) Immune stimulation-inhibition of experimental cancer metastasis. *Cancer Res.*, **34**, 491.

Fidler, I. J., Gersten, D. M. & Kripke, M. L. (1979) Influence of immune status on the metastasis of three murine fibrosarcomas of different immunogenicities. *Cancer Res.*, **39**, 3816.

Fisher, B. & Fisher, E. R. (1967) The organ distribution of disseminated $^{51}Cr$-labelled tumor cells. *Cancer Res.*, **27**, 412.

Gasic, G. J., Gasic, T. B. & Stewart, C. C. (1968) Antimetastatic effects associated with platelet reduction. *Proc. Natl Acad. Sci. U.S.A.*, **61**, 46.

Gasic, G. J., Gasic, T. B., Galanti, N., Johnson, T. & Murphy, S. (1973) Platelet–tumor cells interactions in mice. The role of platelets in the spread of malignant diseases. *Int. J. Cancer*, **11**, 704.

Gastpar, H. (1974) The inhibition of cancer cell stickiness by the methylxanthine derivative pentoxifylline. *Thromb. Res.*, **5**, 277.

Gordon, M. Y., Aguado, M. & Blackett, N. M. (1977) Effects of BCG and *Corynebacterium parvum* on the hematopoietic precursor cells in continuously irradiated mice: Possible mechanisms of action in immunotherapy. *Eur. J. Cancer*, **13**, 229.

Ishibashi, T., Harada, Y., Yamada, H., Harada, S., Takamoto, M. & Sugiyama, K. (1977*a*) Comparison of the mode of immunopotentiating action of BCG and wax D. I. Effect on the immune response to SRBC. *Jap. J. Exp. Med.*, **47**, 163.

Ishibashi, T., Yamada, H., Harada, S., Harada, Y., Takamoto, M. & Sugiyama, K. (1977*b*) Comparison of the mode of immunopotentiation of BCG and wax D. II. Effect on the methylcholanthrene carcinogenesis. *Jap. J. Exp. Med.*, **47**, 435.

Ishibashi, T., Yamada, H., Harada, S., Harada, Y., Takamoto, M. & Sugiyama, K. (1978*a*) Inhibition and promotion of tumor growth by BCG: Evidence for stimulation of humoral enhancing factors by BCG. *Int. J. Cancer*, **21**, 67.

Ishibashi, T., Harada, Y., Harada, S., Yamada, H., Takamoto, M. & Sugiyama, K. (1978*b*) Mode of immunopotentiating action of BCG: Persistence and spread of BCG injection. *Jap. J. Exp. Med.*, **48**, 227.

Miller, T. E., Mackaness, G. B. & Lagrange, P. H. (1973) Immunopotentiation with BCG. II. Modulation of the response to sheep red blood cells. *J. Natl Cancer Inst.*, **51**, 1669.

Müller, R., Lehrach, F. & Grigoleit, H. G. (1975) On the mode of action of pentoxifylline. *Med. Monatsschr.*, **29**, 487.

Nicholson, G. L. & Winkelhake, J. L. (1975) Organ specificity of blood-borne tumor metastasis determined by cell adhesion? *Nature*, **255**, 230.

Prehn, R. T. (1972) The immune reaction as a stimulator of tumor growth. *Science*, **176**, 170.

Schenkein, I., Levy, M. & Uhr, J. W. (1972) The use of glucose oxidase as a generator of $H_2O_2$ in the enzymatic radioiodination of components of cell surfaces. *Cell. Immunol.*, **5**, 490.

Wood, S., Jr (1958) Pathogenesis of metastasis formation observed *in vivo* in the rabbit ear chamber. *Arch. Pathol.*, **66**, 550.